# Vitrimer Chemistry Assisted Fabrication of Aligned, Healable, and Recyclable Graphene/Epoxy Composites

**DOI:** 10.3389/fchem.2019.00632

**Published:** 2019-09-13

**Authors:** Jingjing Chen, Hong Huang, Jinchen Fan, Yan Wang, Junrong Yu, Jing Zhu, Zuming Hu

**Affiliations:** ^1^State Key Laboratory for Modification of Chemical Fibers and Polymer Materials, College of Material Science and Engineering, Donghua University, Shanghai, China; ^2^College of Biological, Chemical Sciences and Engineering, Jiaxing University, Jiaxing, China; ^3^Shanghai Key Laboratory of Materials Protection and Advanced Materials in Electric Power, Shanghai Engineering Research Center of Energy-Saving in Heat Exchange Systems, Shanghai University of Electric Power, Shanghai, China

**Keywords:** graphene nanoplate, vitrimer, composite, alignment, reinforcement

## Abstract

The alignment is a key factor to fully exploit the potential of graphene in reinforcement of polymer composites. However, it is still a challenge to orientate graphene in thermosets because of the insoluble and infusible features of the later. In this paper, we report a facile and scalable hot press method to fabricate aligned graphene nanoplate (GnP)/epoxy composites by utilizing the dynamic character of epoxy vitrimer. The bond exchange and topological rearrangement associated viscous flow of epoxy vitrimer during hot press allows the spontaneous orientation of GnP in matrix because the 2D structure and volume exclusion effect. SEM images demonstrate the orientation of GnP, while tensile test reveals the significantly increased reinforcement effect of GnP on matrix after hot press. Moreover, the dynamic reaction of epoxy vitrimer confers good healability and recyclability to the aligned composites as confirmed by the nearly fully recovered mechanical properties of the healed sample after cutting, and the recycled sample after grinding. This work is expected to provide new opportunity for fabrication of aligned thermosetting composites.

## Introduction

Graphene has been recognized as ideal nanofiller for polymer composites due to its excellent mechanical property, high thermal, and electrical conductivity, and large aspect ratio (Lee et al., 2012; Xin et al., 2014; Shen et al., 2016). To fully realize the potential of graphene in polymer composites, several aspects should be taken into consideration. The exfoliation of graphene into few-layered or even single-layered sheets, the homogeneous dispersion of graphene in matrix, and the strong interfacial interaction between graphene and matrix are generally considered to be the crucial factors that influence the final performance of composites (Terrones et al., 2011; Georgakilas et al., 2012; Punetha et al., 2017; Shi et al., 2018). Although these issues can be addressed by surface modification of graphene, the mechanical improvements in composites achieved so far are always much lower than theoretical values. This situation highlights the importance of another factor, that is, alignment of graphene, in reinforcement of matrix, because the orientation is greatly beneficial for exploitation of the high in-plane mechanical and conductive properties of graphene in composites. Several approaches, such as layer-by-layer assembly (Beese et al., 2014; Xiong et al., 2016; Xie et al., 2018), vacuum-assisted filtration (Xu et al., 2009; Song et al., 2016), hot press (Huang et al., 2012, 2014; Ding et al., 2015), and self-alignment of graphene sheets have been adopted to fabricate aligned graphene/polymer composites (Yousefi et al., 2012; Li Y. et al., 2014; Kumar et al., 2016; Zhao et al., 2016), and it is verified in these studies that the aligned composites are indeed superior to randomly distributed composites in terms of the high-level mechanical reinforcement in orientated direction (Morimune et al., 2014), the anisotropic conductivity (Zhao et al., 2016), and the barrier properties (Li Y. et al., 2014).

Thermosets, represented by epoxy resins, are cross-linked polymers with good mechanical/thermal properties, chemical resistance, and dimensional stability. Combining the merits of thermosets with aligned carbon nanofillers could potentially produce advanced composites with wide applications in aerospace, transportation, building, and other structural materials. However, it is a non-trivial task to orientate graphene in thermosets because of the insoluble and infusible features of the later, which means that most of the abovementioned orientation approaches, such as the simplest and scalable hot press method, can not be applied in these composites. Recently, an aligned graphene/epoxy composite prepared by a self-alignment method has been reported (Yousefi et al., 2013, 2014). The ultralarge graphene sheets were first mixed with the aqueous emulsion of epoxy monomer, then these sheets were self-aligned in matrix during evaporation and curing process due to the “excluded volume” effect. However, the concentration of graphene in matrix must be higher than a critical value, and the preparation process of ultralarge graphene sheets is relatively complicated. Electrical or magnetical field assisted orientation of graphene or magnetic particles decorated graphene in epoxy monomer followed by curing have also been reported, but these strategies suffer from high cost and limited scalability (Yan et al., 2014; Li et al., 2015; Liu et al., 2015, 2016; Renteria et al., 2015; Wu et al., 2015, 2016). Li et al. reported an approach by infiltration of epoxy monomer in aligned and porous graphene paper followed by curing, however, the large fraction of graphene in composites has significantly changed the inherent properties of epoxy resins, and seriously deteriorated the mechanical property of composites (Li Q. et al., 2014). Therefore, it is highly desirable to develop facile and scalable strategy for alignment of graphene in thermoset composites with improved thermal/mechanical properties.

**Figure 1:**
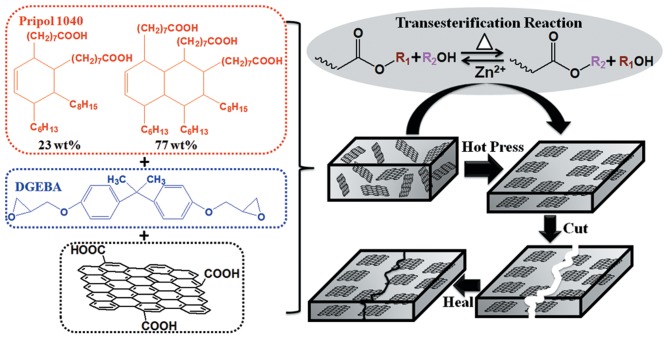
Chemical structure of monomers and the fabrication process of aligned and healable graphene/epoxy composites.

Herein, we report the fabrication of aligned graphene/epoxy composites by a simple hot press method assisted by vitrimer chemistry. Vitrimer is an innovative thermoset that contains exchangeable bonds, such as disulfide bonds (Lei et al., 2014; Ma et al., 2017; Fortman et al., 2018; Huang et al., 2018), imine bonds (Taynton et al., 2014; Lei et al., 2015), ester/hydroxyl groups (Montarnal et al., 2011; Capelot et al., 2012; Lu et al., 2017; Tran et al., 2018), in cross-linked network. It behaves like traditional thermosets with good inherent properties, but can undergo bond exchange reaction and topological rearrangement under certain stimuli (Denissen et al., 2016). These fascinating features endow vitrimers with capability of reshaping, self-healing, and recycling. As such, vitrimers have been adopted in many technological fields that are not applicable by traditional thermosets. For instance, an epoxy vitrimer has been used in 3D printing for recyclable thermosets with complicated geometries (Shi et al., 2017); epoxy and other vitrimers have also been employed to fabricate recyclable fiber reinforced polymer composites (de Luzuriaga et al., 2016; Taynton et al., 2016; Yu et al., 2016; Denissen et al., 2018) Here we demonstrate for the first time the employment of a transesterification based epoxy vitrimer for the fabrication of aligned graphene/epoxy composites by a simple hot press method. During the hot press process, the chain motion and bond exchange associated topological rearrangement were activated, and the whole network exhibited temperature dependent viscous flow behavior, the graphene sheets were then simultaneously orientated perpendicular to the applied force because of their 2D structure and volume exclusion effect (Yousefi et al., 2012; Kumar et al., 2016), as shown in Figure 1. In addition, because of the dynamic property of the matrix, these aligned composites also demonstrated good self-healing and recycling abilities.

## Materials and Methods

### Materials

Graphite intercalation compound (GIC, 100 mesh, 99.5%) was purchased from Ao Yu Co. Ltd in Shanghai. Bisphenol A diglycidyl ether (DGEBA) was purchased from TCI Co. Ltd in Shanghai. Fatty acid (Pripol 1040) was supplied by He Da chemical Co. Ltd (Shanghai). Zinc acetate dihydrate, acetone, ethanol, concentrated nitric acid, and concentrated sulfuric acid were obtained from Sinopharm Chemical Reagent Co. Ltd. All chemicals were used as received without further treatment.

### Preparation of Graphene Nanoplate (GnP)

Expanded graphite (EG) was first obtained by thermal expansion of GIC at 700°C for 1 min. Then 1 g EG was refluxed in 100 mL mixture of concentrated nitric acid and concentrated sulfuric acid (volume ratio of 1:3) at 100°C for 4.5 h to afford acidic EG (EG-COOH). At last, 1 g EG-COOH was suspended in 100 mL ethanol and sonicated in a low power sonic bath for 24 h followed by centrifugation at 1,000 rpm for 30 min, the supernatant containing exfoliated GnP was collected, and the concentration was calculated by drying a certain amount of the dispersion of GnP.

### Preparation of GnP/Epoxy Composites

The procedure for the fabrication of epoxy and GnP/epoxy composites were similar to previously reported methods (Montarnal et al., 2011; Yu et al., 2016). Fatty acid and zinc ions catalyst (the molar ratio between carboxyl groups and zinc ions was 1:0.05) were first mixed together and heated at 180°C for 2–3 h in vacuum to dissolve the zinc ions. Then required amount of DGEBA (with the stoichiometric amount of epoxy and carboxyl groups) and GnP suspension were added in the mixture. After homogeneously mixing of these compounds, the solvent was completely removed by rotary evaporator. The precursors were then poured into a mold (70 × 10 × 3 mm) and cured at 130°C for 6 h. The content of GnP in composites were set as 0.5, 1, 2, and 3 wt%, and the as-prepared composites were denoted as aGnP/epoxy.

For the orientation of GnP in composites, the aGnP/epoxy were placed between two metal plate of a home-made tablet press, and preheated at 200°C for 20 min, then the specimens were compressed to 1 mm and 0.5 mm in thickness (compression ratio of 67 and 83%). After maintained at 200°C for another 5 min, the specimens were cooled to room temperature. The hot pressed composites were then denoted as hGnP/epoxy.

### Characterizations

Fourier transform infrared (FT-IR) spectra were obtained from a Nicolet 6,700 spectrometer. Transmission electron microscopy (TEM) images were obtained using JEM-2100. Atomic force microscopy (AFM) images were recorded using a digital Nanoscope IIIa Atomic Force Microscope in tapping mode. Rheological test was performed on ARES-RFS rheometer in a strain-controlled mode, the sample was in disk-shape with diameter of 8 mm and thickness of 2 mm. From the strain sweep experiments at a frequency of 1 rad/s for determination of the region of linear response, the strain of 0.4% was applied for stress relaxation experiments and the relaxation modulus was recorded as a function of time at different temperatures. Differential scanning calorimetry (DSC) test was performed on a Netzsch 204 F1 thermal analyzer from −20 to 80°C at a heating rate of 10°C/min under nitrogen flow. Thermogravimetric analysis (TGA) was performed with a Netzsch TGA 209 F1 instrument at a heating rate of 20°C/min in nitrogen. Tensile tests were measured using MTS materials testing machine at room temperature and humidity of about 50%, the gauge length was 30 mm and strain rate was 50 mm/min, at least five specimens of each sample were measure for accuracy.

## Results and Discussion

### Synthesis of aGnP/Epoxy Composites

GnP was prepared by acidification and solvent exfoliation, which is similar to previously reported process (Tian et al., 2013). The FT-IR spectra (Supplementary Figure 1a) reveal the appearance of characteristic peak of carboxyl group at 1,736 cm^−1^ in EG-COOH, while TGA curves (Supplementary Figure 1b) suggest the weight loss of EG-COOH at 600°C is increased to 26.6%, demonstrating the generation of carboxyl groups in EG-COOH after acid treatment. The increased intensity ratio of D band to G band of EG-COOH as compared to that of EG in Raman spectra (Supplementary Figure 1c) also verifies the introduction of functional groups. Note that the carboxyl groups in EG-COOH could not only promote its exfoliation in polar solvents, but also allow the covalent bonding of GnP with matrix and the participating of GnP in transesterification reaction, which is beneficial to the stress relaxation of the whole system (Legrand and Soulié-Ziakovic, 2016; Tang et al., 2017). GnP was obtained by exfoliation of EG-COOH in ethanol. TEM and AFM characterizations (Supplementary Figure 2) confirm the successful exfoliation of GnP from EG-COOH with thickness ranging from 3~5 nm, corresponding to 10~16 layers. The elastomeric epoxy matrix is cured from DGEBA and fatty acid in the presence of zinc ions as catalyst, which is the same as previously used epoxy vitrimer for other applications (Montarnal et al., 2011; Yu et al., 2016; Shi et al., 2017). FT-IR spectra (Supplementary Figure 3) demonstrate the curing of epoxy resin, while rheological test (Supplementary Figure 4) reveals the stress relaxation behavior of epoxy resin at high temperature, corresponding to the occurrence of bond exchange and topological rearrangement. The fitting of relaxation times at different temperatures indicates the apparent activation energy of epoxy resin is 90 ± 6 kJ mol^−1^, which is similar to previously reported values (Montarnal et al., 2011; Yu et al., 2016; Shi et al., 2017).

**Figure 2:**
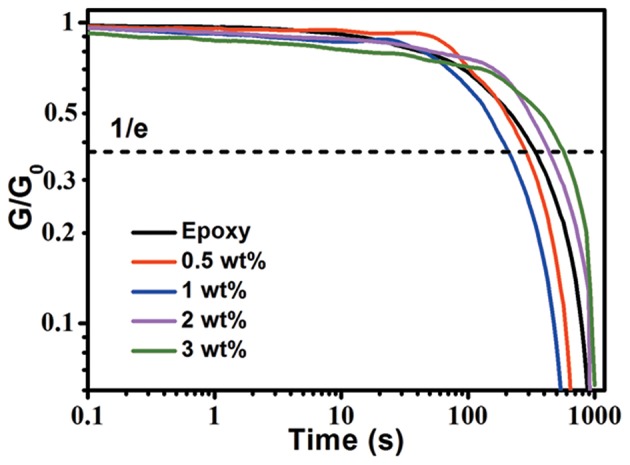
Normalized stress relaxation curves of epoxy and aGnP/epoxy composites at 200°C.

The aGnP/epoxy composites were then fabricated by mixing GnP with epoxy monomers followed by thermal curing. The effect of GnP on the dynamic behavior of epoxy was inspected by rheological test in order to determine the optimal condition for hot press of aGnP/epoxy composites. As shown in Figure 2, the relaxation time (τ^*^, defined as the time for stress relaxation modulus to reach 1/e) of epoxy at 200°C is 346 s. The addition of 0.5 and 1 wt% GnP decreases the τ^*^ to 288 and 206 s, suggesting the accelerated stress relaxation, which is attributed to the decreased cross-linking density because of the introduced excess amount of carboxyl groups in the cross-linked network, and the surface exchangeable bonds on GnP after curing that could participate in the relaxation process of the network (Legrand and Soulié-Ziakovic, 2016). However, further increasing of GnP to 2 and 3 wt% inversely increases the τ^*^ to 443 and 568 s, indicating the restricted chain mobility by GnP has hindered the stress relaxation of the whole network, and the restriction effect has surpassed the abovementioned positive effects at higher loadings of GnP. Nevertheless, all composites could fully relax within 1000 s at 200°C, proving the possibility of fabricating aligned GnP/epoxy composites by hot press.

### Characterizations of Aligned hGnP/Epoxy Composites

**Figure 3:**
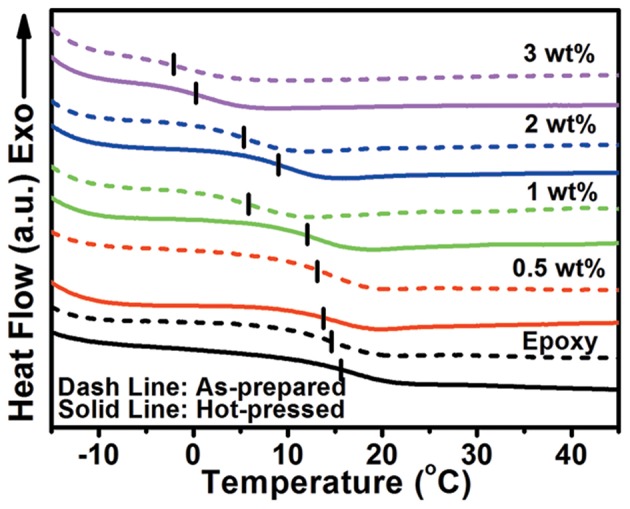
DSC curves of as-prepared and hot-pressed GnP/epoxy composites.

The thermal and mechanical properties of hGnP/epoxy composites (compression ratio of 67%) were then investigated. Figure 3 presents the DSC curves of aGnP/epoxy and hGnP/epoxy composites. The glass transition temperature (*T*_*g*_) of epoxy and composites are summarized in Table 1. The *T*_*g*_ decreases with the addition of GnP due to the reduced cross-linking density of the whole network. The variation tendency of *T*_*g*_ of hGnP/epoxy composites is similar to that of aGnP/epoxy composites. However, in comparison with their corresponding aGnP/epoxy samples, the *T*_*g*_ of hGnP/epoxy composites are all increased. The slightly increased *T*_*g*_ in hot-pressed epoxy (Δ*T*_*g*_ = 0.3°C) is ascribed to the more compacted molecular chains after hot press (Li et al., 2018). While the Δ*T*_*g*_ in hGnP/epoxy composites is elevated with the increasing loading of GnP, suggesting the pressure induced confinement of polymer between GnP layers has restricted their thermal motion more effectively (Wang et al., 2019). The thermal stability of all composites were also investigated by TGA, as shown in Supplementary Figure 5. It is found that the addition of GnP or the hot press have negligible effect on the thermal stability of composites.

**Table 1:** Physical properties of aGnP/epoxy and hGnP/epoxy composites.

	***T_***g***_* (**°**C)**	***E* (MPa)**	**σ (MPa)**	**ε (%)**
Epoxy^a^	14.6	1.8 ± 0.2	3.0 ± 0.2	143.2 ± 12.5
0.5 wt%^a^	13.2	1.9 ± 0.2	3.5 ± 0.4	170.6 ± 15.4
1 wt%^a^	5.8	2.0 ± 0.3	3.7 ± 0.4	245.3 ± 19.2
2 wt%^a^	5.4	0.8 ± 0.2	2.2 ± 0.2	220.4 ± 17.3
3 wt%^a^	−2.1	0.5 ± 0.1	1.4 ± 0.1	263.5 ± 20.4
Epoxy^b^	15.7	2.3 ± 0.3	3.5 ± 0.3	148.7 ± 15.3
0.5 wt%^b^	14.1	2.2 ± 0.2	6.0 ± 0.5	280.8 ± 25.2
1 wt%^b^	12.0	2.8 ± 0.3	6.9 ± 0.6	306.4 ± 23.7
2 wt%^b^	8.9	2.5 ± 0.4	5.3 ± 0.3	245.7 ± 18.5
3 wt%^b^	0.5	1.6 ± 0.2	3.3 ± 0.3	211.6 ± 20.6
1 wt%^c^	–	3.0 ± 0.3	8.2 ± 0.4	237.8 ± 17.8
3 wt%^c^	–	2.2 ± 0.2	5.5 ± 0.3	297.6 ± 28.7

a *As-prepared*.

b *Hot-pressed with compression ratio of 67%*.

c *Hot-pressed with compression ratio of 83%*.

**Figure 4:**
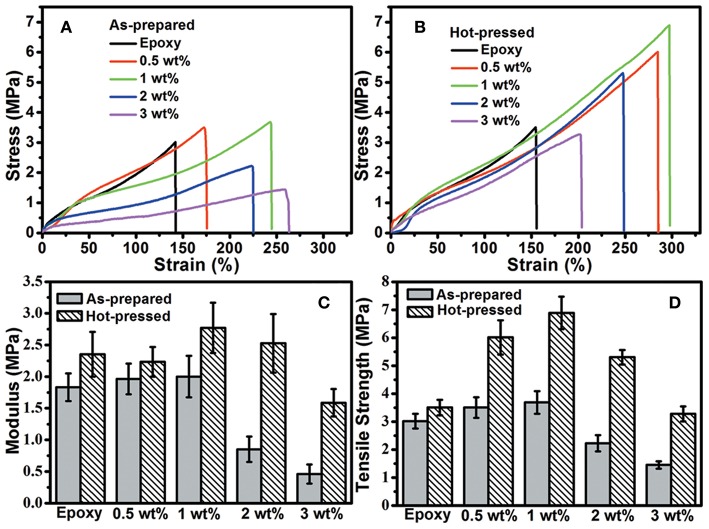
Typical stress-strain curves of **(A)** aGnP/epoxy and **(B)** hGnP/epoxy composites. The comparisons of **(C)** modulus and **(D)** tensile strength of aGnP/epoxy and hGnP/epoxy composites with the same loading of GnP.

The mechanical properties of hGnP/epoxy composites were evaluated by tensile test and compared to those of aGnP/epoxy composites, as shown in Figure 4 and Table 1. It is found that the addition of GnP has moderately improved the modulus (*E*) and tensile strength (σ) of epoxy. The aGnP/epoxy with 1 wt% GnP shows the highest modulus and tensile strength of 2.0 and 3.7 MPa, corresponding to increments of 11.1 and 23.3% as compared to epoxy, respectively. The elongation at break (ε) of composites are also increased as a result of the decreased cross-linking density. Further addition of GnP to higher than 2 wt% has lowered the *E* and σ of composites, which is probably due to the negative effect of lowered cross-linking density has overwhelmed the positive effect of mechanical reinforcement of GnP. As expected, the hot press has significantly enhanced the reinforcement effect of GnP on epoxy. For example, the *E* and σ of hGnP/epoxy with 1 wt% GnP is promoted to 2.8 and 6.9 MPa, which are 40.0 and 86.5% higher than its corresponding aGnP/epoxy composite, and 55.6 and 130.0% higher than epoxy. The hGnP/epoxy with 2 wt% GnP exhibits more enhancement in *E* and σ (with increments of 194 and 141% as compared to its aGnP/epoxy composite) after hot press. Note that the *E* and σ of pure epoxy are also improved by hot press due to the more compacted molecular chains (Li et al., 2018), but the increments in *E* and σ (27.8 and 16.7 %) are much lower than those in composites. In addition, it is also noted that there might exist additional esterification reaction between excess carboxyl groups and hydroxyl groups in matrix during hot press, which could also contribute to the improved mechanical properties of hGnP/epoxy composites, but a comparison made on non-dynamic composites (Supplementary Figure 6) using the same hot press procedure suggest negligible improvements in tensile properties. These comparisons indicate that the improvements in mechanical properties of composites are mainly resulted from the orientation of GnP in matrix, but not from the compact molecular chains or increased cross-linking after hot press.

**Figure 5:**
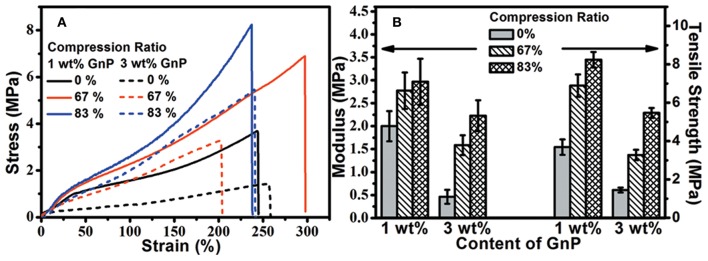
**(A)** Typical stress-strain curves of hGnP/epoxy composites with difference compression ratio. **(B)** Comparisons of the modulus and tensile strength of hGnP/epoxy composites with difference compression ratio.

The influence of compression ratio on mechanical properties of composites were also evaluated by tensile test using composites with 1 and 3 wt% GnP as examples. Figure 5A shows the typical stress-strain curves of composites with different compression ratio and Figure 5B compares their *E* and σ. It can be seen that increase of the compression ratio from 67 to 83% further improves the mechanical properties of composites. Notably, hGnP/epoxy with only 1 wt% GnP and compression ratio of 83% exhibits strength of 8.2 MPa, which is 173.3% higher than epoxy, suggesting the higher degree of compression could lead to more significant reinforcement of GnP on epoxy, which might be due to the better orientation of GnP with higher compression ratio.

**Figure 6:**
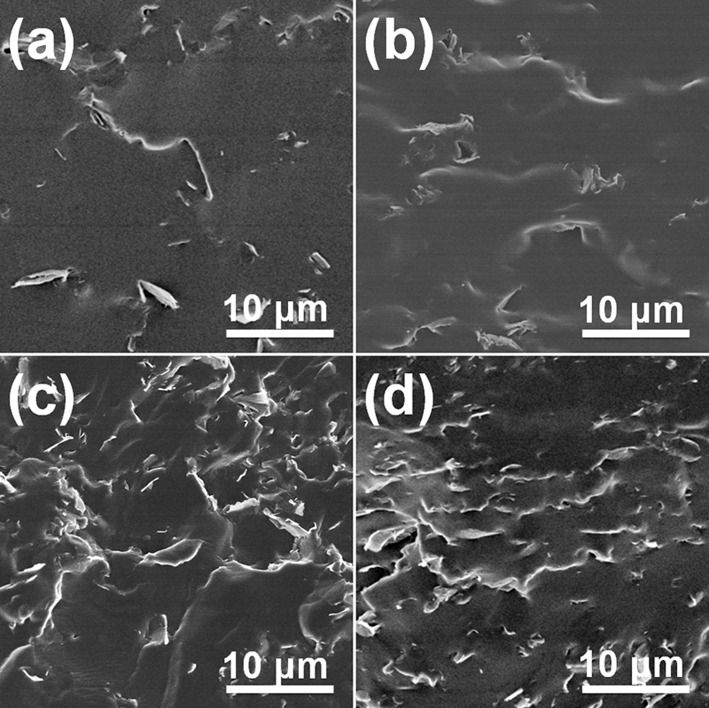
SEM images of aGnP/epoxy with **(a)** 1 wt% and **(c)** 3 wt% GnP, and hGnP/epoxy with **(b)** 1 wt% and **(d)** 3 wt% GnP.

SEM images of cross sections of aGnP/epoxy and hGnP/epoxy (compression ratio of 83%) composites were then employed to directly demonstrate the orientation of GnP in epoxy matrix by hot press (Figure 6). It can be clearly seen the randomly distributed GnP in aGnP/epoxy with 1 and 3 wt% GnP, while the edges of GnP are aligned in hGnP/epoxy composites after hot press, corresponding to the orientation of GnP layers in matrix. This observation confirms our assumption that the hot press could orientate the GnP in dynamic epoxy and greatly promote the reinforcement effect.

We have also carried out control experiments on non-dynamic GnP/epoxy composites to highlight the important of transesterification reaction in the hot press process. The non-dynamic composites were prepared from the same raw materials and procedure but without the addition of zinc ions as catalyst. The tensile test (Supplementary Figure 6) reveals that the hot press has only little impact on the mechanical properties of non-dynamic composites regardless of the loading of GnP or the compression ratio. This is because in non-dynamic network, even if the applied force could induce the macroscopic deformation of the network at temperature higher than *T*_*g*_, and the deformed shape could be fixed at lower temperature, the internal stress generated by the external force could not be fully released because the restricted chain mobility by covalent cross-linking points. Such stored stress is harmful to the dimensional stability and mechanical properties of network. Moreover, once these deformed samples were used in some high-temperature applications, the high temperature would activate the frozen polymer chains, thus the deformed sample are tend to recover its original shape, such phenomenon is well-known as “shape memory effect” (Rousseau and Xie, 2010; Zheng et al., 2015). On the contrary, the dynamic reaction in our vitrimer composites could allow the full release of internal stress by bond exchange and chain rearrangement, thus permits the orientation of GnP in response to viscous flow of the network, and the achieving of stable and highly reinforced aligned GnP/epoxy composites.

### Healability and Recyclability of Aligned hGnP/Epoxy Composites

**Figure 7:**
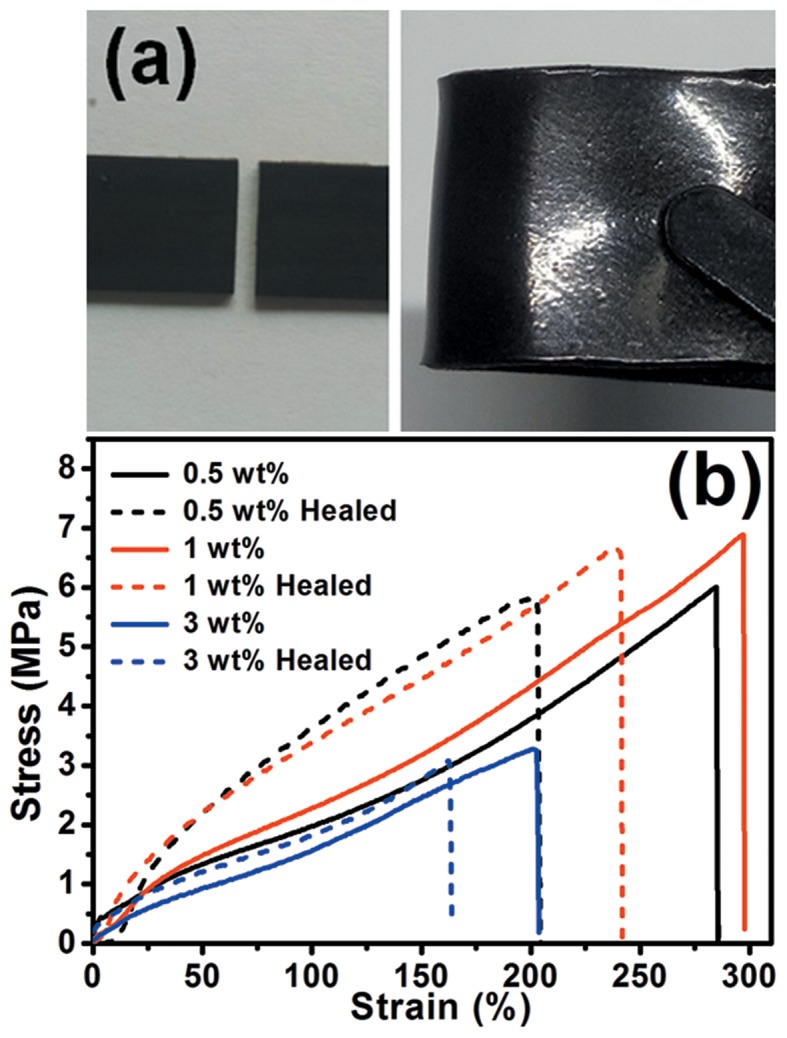
**(a)** Photographs of fractured and healed hGnP/epoxy with 1 wt% GnP. **(b)** Typical stress-strain curves of original and healed hGnP/epoxy composites.

The dynamic transesterification reaction not only allows the fabrication of aligned hGnP/epoxy composites by simple hot press process, but also confers healability and recyclability to the composites because of the capability of bond exchange. To demonstrate these points, the specimens of hGnP/epoxy were first cut in half, then the fracture surfaces were put together under slight pressure and heated at 200°C for 20 min. As shown in Figure 7a, the broken specimens were joint together without obvious scar. Tensile test was used to quantitatively evaluated the healability of hGnP/epoxy composites (Figure 7b). The healing efficiency (by using recovery in strength as criterion) of hGnP/epoxy with 0.5, 1, and 3 wt% GnP are detected to be 97.1, 96.8, and 95.1%, respectively, demonstrating the good healability of our hGnP/epoxy. Moreover, it is found that the healing efficiency of aGnP/epoxy with 0.5, 1, and 3 wt% GnP are 97.4, 97.8, and 96.0%, respectively (Supplementary Figure 7), which are similar to those of hGnP/epoxy, suggesting the orientation of GnP in epoxy has negligible effect on the healing performance of composites.

**Figure 8:**
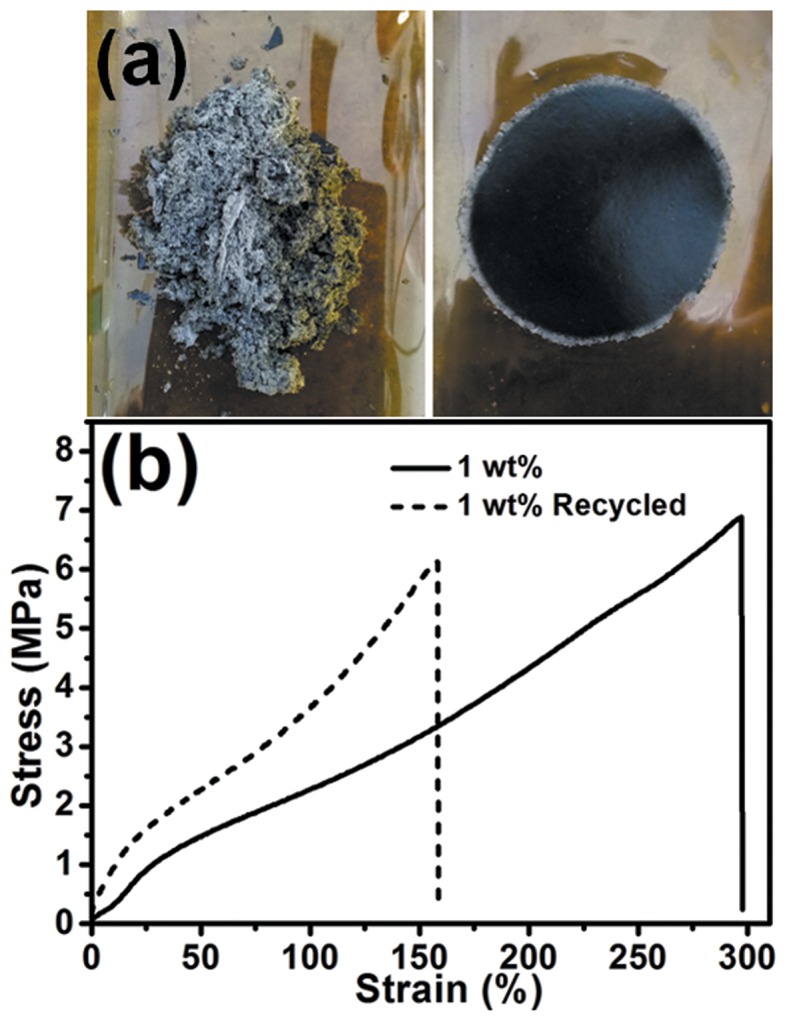
**(a)** Photographs of grinded and recycled hGnP/epoxy with 1 wt% GnP. **(b)** Typical stress-strain curves of original and recycled hGnP/epoxy with 1 wt% GnP.

To verify the recyclability of hGnP/epoxy composites, the composite with 1 wt% GnP was first grinded into fine powder, then the powder was hot pressed at 200°C for 30 min, the thickness of the recycled composite was controlled the same as original one. It can be seen that the grinded powder formed integrated composite again after hot press (Figure 8a). The tensile test (Figure 8b) reveals that the σ of recycled composite is 6.2 MPa that is nearly constant with that of original composite (6.9 MPa). While the ε of recycled sample is reduced from 306.4 to 158.8%, which may be due to the aging of composite at high temperature or some irreversible side reactions such as the esterification reaction between excess carboxyl groups and hydroxyl groups as previously observed in other studies (Imbernon et al., 2016; Zhang et al., 2018). Nevertheless, the results confirms the potential of our aligned hGnP/epoxy for use as recyclable materials.

## Conclusions

This work presents a facile and scalable hot press method for the fabrication of aligned GnP/epoxy composites by using a epoxy vitrimer as matrix. The GnP could be simultaneously orientated in matrix during hot press as confirmed by SEM images due to the ability of epoxy vitrimer to viscous flow at high temperature. The tensile test demonstrated that the mechanical properties of composites were greatly improved after hot press, and the improvement was closely related to the compression ratio of hGnP/epoxy composites, larger compression ratio resulted in greater improvement in mechanical properties. It is showed that with the addition of only 1 wt% GnP, the strength of hGnP/epoxy with compression ratio of 83% was 173.3% higher than pristine epoxy, while the increment was just 23.3% in aGnP/epoxy. The comparison with hGnP/epoxy composites with no catalyst revealed the crucial role of vitrimer character in fabrication of aligned composites. In addition, the tensile test showed that the healing efficiency of hGnP/epoxy composites were generally higher than 95% after cutting in half, and the strength of recycled hGnP/epoxy after grinding into powder was nearly constant with that of original value, proving the good healability and recyclability of hGnP/epoxy composites. We anticipate that the approach of fabricating aligned GnP/epoxy composites assisted by vitrimer chemistry reported here could be applied in design of other aligned thermosetting composites.

## Data Availability

All datasets generated for this study are included in the manuscript/Supplementary Files.

## Author Contributions

JC, HH, JF, and YW contributed to the design and synthesis of polymer and composites. JC, JF, YW, JY, and JZ contributed to the characterizations of properties of composites and data analysis. JC, JF, and YW contributed to the writing of the first draft. HH, JF, YW, and ZH contributed to the final revision of the draft.

### Conflict of Interest Statement

The authors declare that the research was conducted in the absence of any commercial or financial relationships that could be construed as a potential conflict of interest.
