# Supplementary material for: Vitrimer Chemistry Assisted Fabrication of Aligned, Healable, and Recyclable Graphene/Epoxy Composites
Source: Front Chem. 2019 Sep 13;7:632. doi: 10.3389/fchem.2019.00632 (PMC6753619; doi:10.3389/fchem.2019.00632)
Supplement: Supplementary file 1 [file Data_Sheet_1.PDF]

Supplementary Material

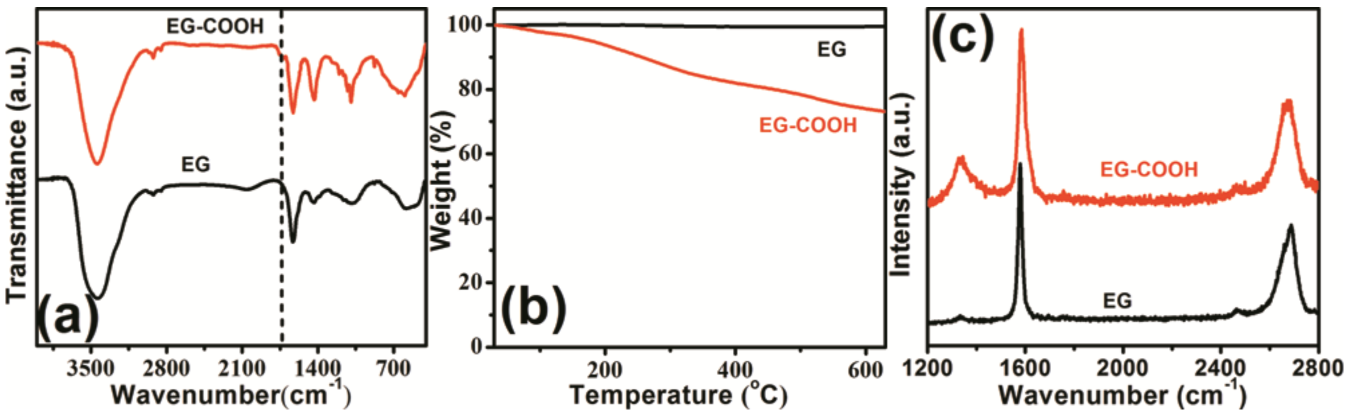

Supplementary Figure 1. (a) FT-IR spectra, (b) TGA curves, and (c) Raman spectra of EG and EG-COOH.

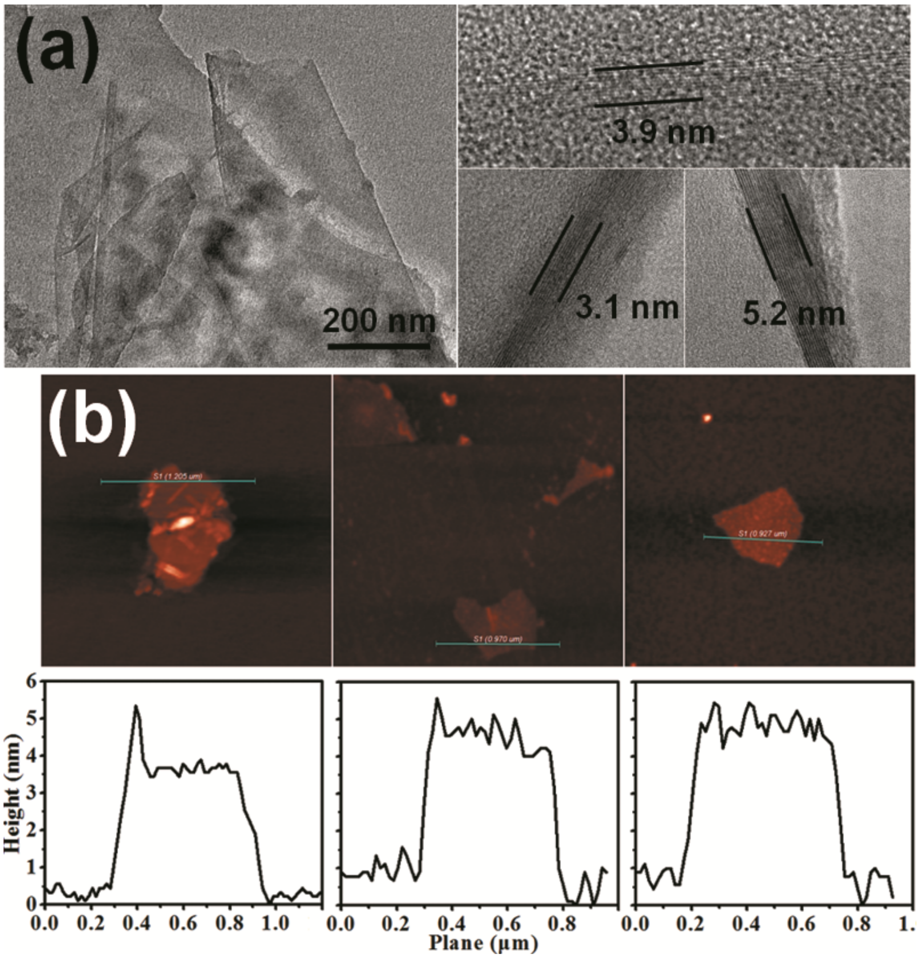

Supplementary Figure 2. (a) TEM images of GnP. (b) AFM images and height profiles of GnP.

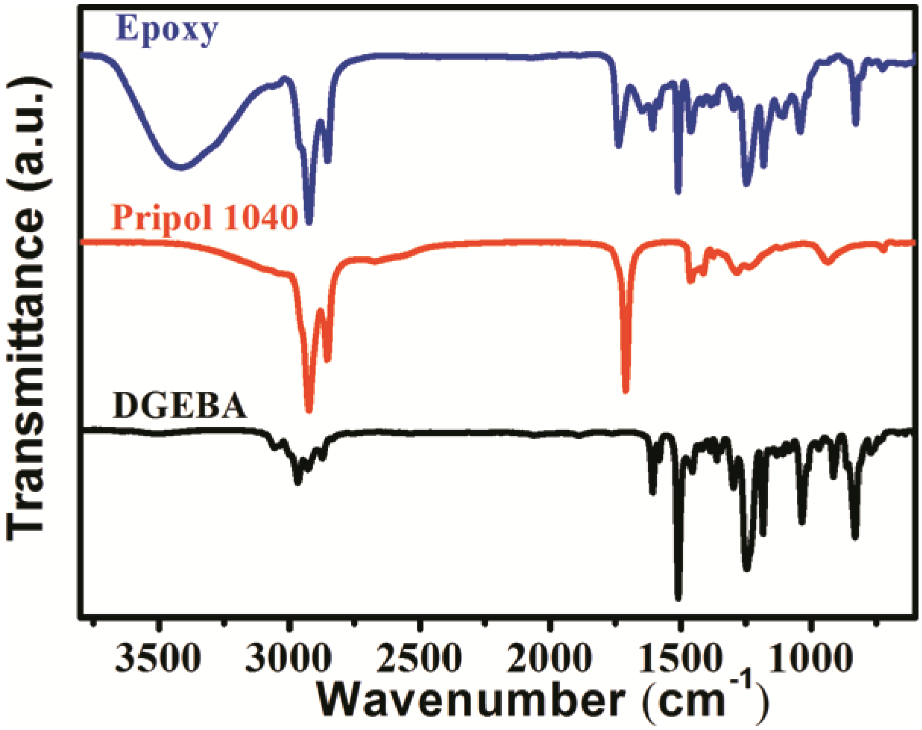

Supplementary Figure 3. FT-IR spectra of DGEBA, pripol 1040, and cured epoxy resin.

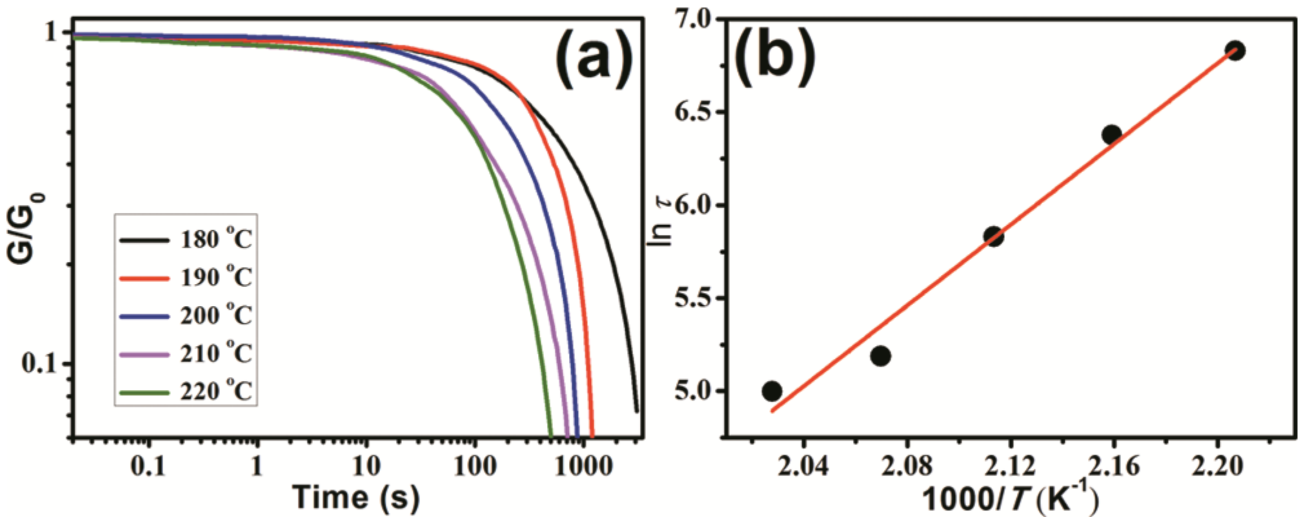

Supplementary Figure 4. (a) Normalized stress relaxation curves of epoxy resin at different temperatures. (b) Fitting of the relaxation times of epoxy resin to the Arrhenius equation.

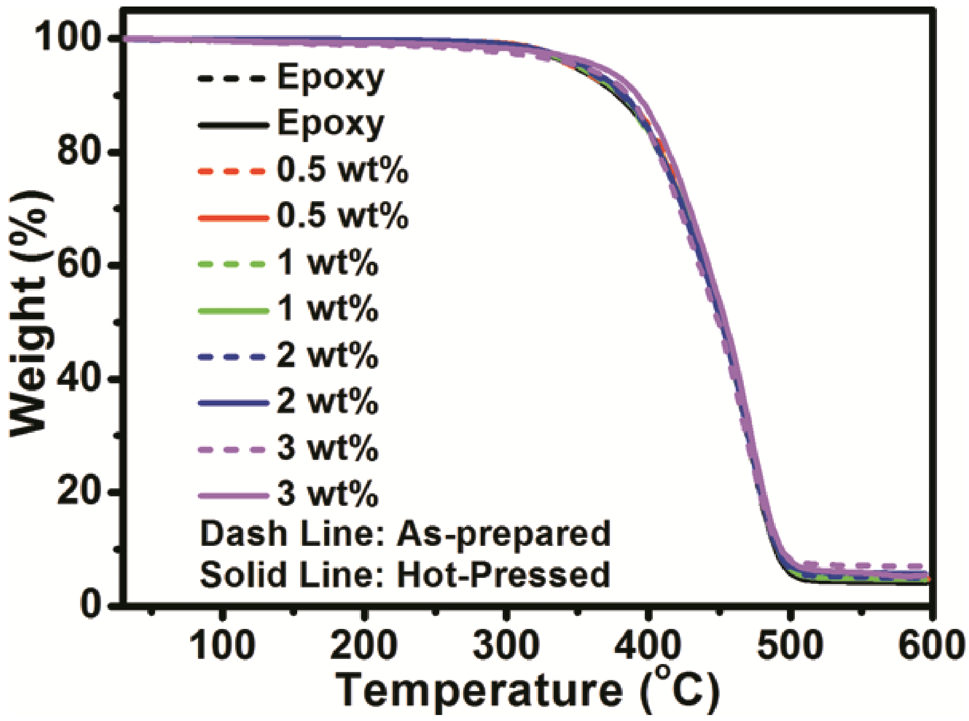

Supplementary Figure 5. TGA curves of aGnP/epoxy and hGnP/epoxy composites.

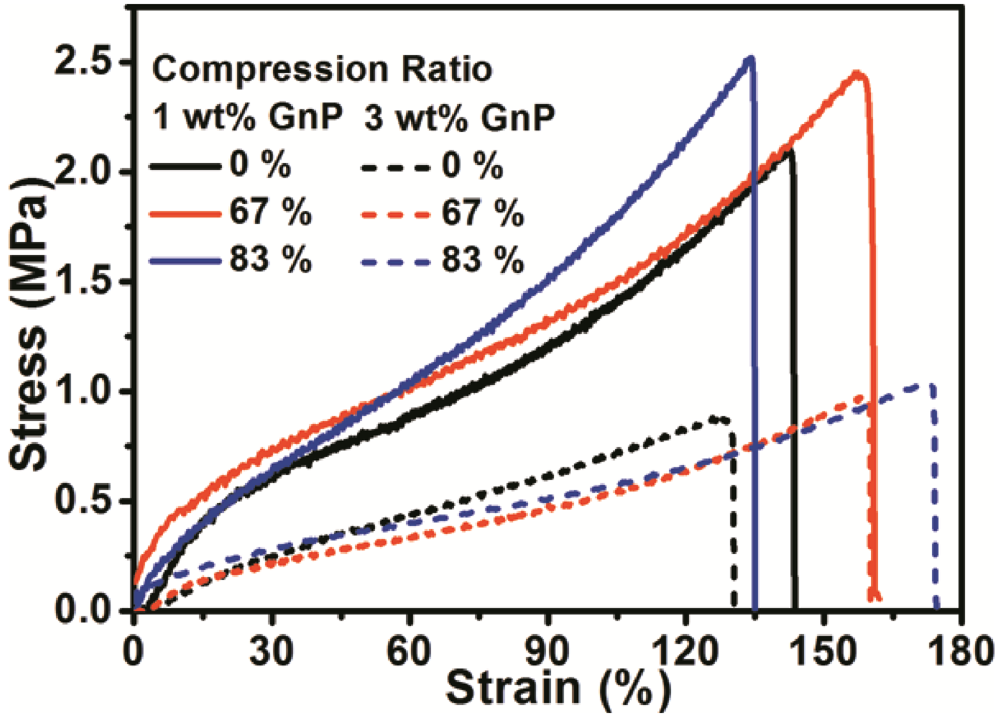

Supplementary Figure 6. Typical stress-strain curves of hGnP/epoxy composites without the addition zinc ions as catalyst.

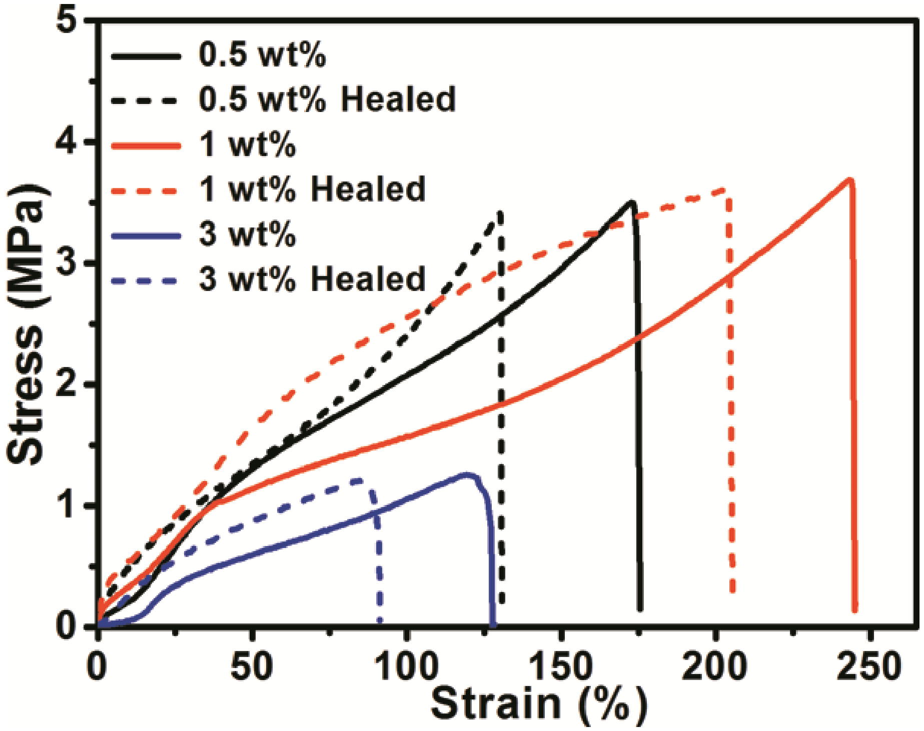

Supplementary Figure 7. Typical stress-strain curves of original and healed aGnP/epoxy composites.
